# From Disease Association to Risk Assessment: An Optimistic View from Genome-Wide Association Studies on Type 1 Diabetes

**DOI:** 10.1371/journal.pgen.1000678

**Published:** 2009-10-09

**Authors:** Zhi Wei, Kai Wang, Hui-Qi Qu, Haitao Zhang, Jonathan Bradfield, Cecilia Kim, Edward Frackleton, Cuiping Hou, Joseph T. Glessner, Rosetta Chiavacci, Charles Stanley, Dimitri Monos, Struan F. A. Grant, Constantin Polychronakos, Hakon Hakonarson

**Affiliations:** 1 Department of Computer Science, New Jersey Institute of Technology, Newark, New Jersey, United States of America; 2 Center for Applied Genomics, The Children's Hospital of Philadelphia, Philadelphia, Pennsylvania, United States of America; 3 Departments of Pediatrics and Human Genetics, McGill University, Montreal, Québec, Canada; 4 Division of Endocrinology, Department of Pediatrics, The Children's Hospital of Philadelphia, Philadelphia, Pennsylvania, United States of America; 5 Department of Pathology and Laboratory Medicine, The Children's Hospital of Philadelphia, Philadelphia, Pennsylvania, United States of America; 6 Division of Genetics, Department of Pediatrics, The Children's Hospital of Philadelphia, Philadelphia, Pennsylvania, United States of America; Queensland Institute of Medical Research, Australia

## Abstract

Genome-wide association studies (GWAS) have been fruitful in identifying disease susceptibility loci for common and complex diseases. A remaining question is whether we can quantify individual disease risk based on genotype data, in order to facilitate personalized prevention and treatment for complex diseases. Previous studies have typically failed to achieve satisfactory performance, primarily due to the use of only a limited number of confirmed susceptibility loci. Here we propose that sophisticated machine-learning approaches with a large ensemble of markers may improve the performance of disease risk assessment. We applied a Support Vector Machine (SVM) algorithm on a GWAS dataset generated on the Affymetrix genotyping platform for type 1 diabetes (T1D) and optimized a risk assessment model with hundreds of markers. We subsequently tested this model on an independent Illumina-genotyped dataset with imputed genotypes (1,008 cases and 1,000 controls), as well as a separate Affymetrix-genotyped dataset (1,529 cases and 1,458 controls), resulting in area under ROC curve (AUC) of ∼0.84 in both datasets. In contrast, poor performance was achieved when limited to dozens of known susceptibility loci in the SVM model or logistic regression model. Our study suggests that improved disease risk assessment can be achieved by using algorithms that take into account interactions between a large ensemble of markers. We are optimistic that genotype-based disease risk assessment may be feasible for diseases where a notable proportion of the risk has already been captured by SNP arrays.

## Author Summary

An often touted utility of genome-wide association studies (GWAS) is that the resulting discoveries can facilitate implementation of personalized medicine, in which preventive and therapeutic interventions for complex diseases can be tailored to individual genetic profiles. However, recent studies using whole-genome SNP genotype data for disease risk assessment have generally failed to achieve satisfactory results, leading to a pessimistic view of the utility of genotype data for such purposes. Here we propose that sophisticated machine-learning approaches on a large ensemble of markers, which contain both confirmed and as yet unconfirmed disease susceptibility variants, may improve the performance of disease risk assessment. We tested an algorithm called Support Vector Machine (SVM) on three large-scale datasets for type 1 diabetes and demonstrated that risk assessment can be highly accurate for the disease. Our results suggest that individualized disease risk assessment using whole-genome data may be more successful for some diseases (such as T1D) than other diseases. However, the predictive accuracy will be dependent on the heritability of the disease under study, the proportion of the genetic risk that is known, and that the right set of markers and right algorithms are being used.

## Introduction

Genome-wide association studies (GWAS) have been successfully employed to interrogate the genetic architecture of common and complex diseases [1]. Unlike traditional linkage and candidate gene association studies, GWAS have enabled human geneticists to examine a wide range of complex phenotypes, and have allowed the confirmation and replication of previously unsuspected susceptibility loci. Some of the more notable examples of success include dozens of susceptibility loci now known to modify individual disease risk of type 2 diabetes (T2D) [2], type 1 diabetes (T1D) [3],[4], Crohn's disease (CD) [5], as well as loci influencing polygenic traits such as height [6]–[8], body mass index [9] and dyslipidemia [10]. However, for many conditions, these variants still explain only a small proportion of individual differences in disease predisposition or phenotypic diversity; for example, the 54 validated loci that influence human height collectively only explain 4–6% of variation in the trait after adjustment of age and sex [11], and the 31 validated susceptibility loci for Crohn's disease collectively only explains 20% of the genetic risk variance [5]. Identifying most of the remaining genetic variance still represents a challenge, albeit tractable, for the foreseeable future.

Besides identifying genes influencing disease susceptibility or phenotypic variation, another often suggested utility of GWAS is that these discoveries will facilitate implementation of personalized medicine, in which preventive and therapeutic interventions for complex diseases are tailored to individuals based on their genetic make-up, as can be determined by genome-wide genotyping profiles on a SNP-based array. The latter promise is now routinely used for certain monogenic disorders where the underlying genetic factor has already been characterized; however, for common and complex diseases, where multiple loci work together to increase disease risk, the application of personalized medicine may not be as straightforward. In fact, several studies have been recently published on the assessment of risk for common diseases using multiple genetic variants (reviewed in [12]–[16]). However, a consistent theme from these studies is that disease assessment methods so far show limited predictive value, and the performance of these studies is far below what would be considered clinically feasible or practical. For example, at least four studies have been conducted to use several “validated” variants for risk prediction of type 2 diabetes (T2D) [17]–[20]. The AUC (area under the receiver operating characteristic curve) scores for the T2D studies range from 0.55 to 0.60, indicating that the prediction is only slightly better than chance; however, they also indicate that the use of more markers should lead to improvement of predictive performance. A more recent study on multiple human diseases reported slightly higher AUC scores for Age-related Macular Degeneration (AMD) and Crohn's Disease (CD), but the results are still largely negative; in fact, the authors cautioned that the scientific community should avoid “overstating the value of association findings in terms of personalized medicine” [15]. Other similar negative studies have been conducted for a variety of human diseases and complex traits, such as height [11], coronary heart disease [21] and cardiovascular diseases [22], although positive studies have been reported for AMD when combining genetic, demographic, and environmental variables [23]. Altogether, these observations have triggered concern about the potential value of individual-based disease risk assessment, at least for the time being.

Our view is that since the majority of risk factors for human diseases or complex traits have yet to be identified, the failure of previous studies on predicting individual disease risk is not unexpected: First, all these studies have investigated only a limited number of susceptibility variants that were confirmed in previous GWAS. As previously discussed, these validated susceptibility loci typically only explain a small proportion of the genetic risk underlying phenotypic variance. Therefore, the omission of the vast majority of genuine susceptibility loci that are yet to be validated from GWAS precludes success, since most of the informative markers are absent from prediction model. For example, none of the four T2D risk assessment studies [17]–[20] used more than 20 SNPs; we do not expect that any such study would yield satisfactory results from a handful of loci, which collectively explain only a minor fraction of disease risk. We however acknowledge that the reason why most genetic variants have not been identified is because the first generation of GWAS were powered to detect variants of large effect sizes; with the ever increasing sample sizes in GWAS, more loci will be discovered and validated in the future. Second, only relatively simple statistical approaches, such as additive genotype scores (unweighted or weighted number of risk alleles) or logistic regression assuming independence between variants, have been applied in previous studies. These approaches, although widely used in statistical genetics, do not take into account the complex relationships or interactions between multiple loci contributing to disease risk. In fact, regression analysis is optimized for the purpose of estimating the effects of predictor variables, unlike other more sophisticated machine-learning approaches (especially maximum-margin approaches) for the sole purpose of classification or discrimination. Finally, the whole-genome genotype data in the diseases from these previous studies are probably “overly” complex, with the genetics *per se* possibly only explaining a small proportion of disease incidence, unless coupled with other factors such as environmental exposures. For example, T2D has a heritability estimate of ∼50% [24] while T1D has a much stronger familial component, with a heritability estimate of ∼90% [25]. Therefore, we expect that whole-genome genotype-based disease risk assessment would operate better in T1D than in previous studies of T2D. Altogether, we are not proposing that the failure of previous studies indicates that disease risk assessment is infeasible, rather that alternative routes should be taken for a better evaluation of individual disease risk assessment.

In the current study, we have attempted to address the issues discussed above. First, rather than cherry-picking a few known susceptibility loci for disease risk assessment, we utilized an entire list of markers reaching a pre-defined statistical threshold for association with a disease (for example, *P*<1×10^−5^), even if the majority of SNPs in that list have not been confirmed to be genuine susceptibility loci. There is no doubt that some false positive hits will arise, but we show that the computational approaches used are in fact robust with the inclusion of these non-contributing markers when also taking advantage of other markers that are already established to be associated with the disease. Second, we have utilized Support Vector Machine (SVM) [26], a well-developed machine-learning technique in computer science. Unlike traditional “number of risk alleles” approach or logistic regression assuming independence between markers, our approach can both optimize prediction modeling and take advantage of potential interactions between markers to achieve the optimal binary predictive power. Finally, we have used T1D as an example of our efforts for disease assessment. Unlike other common diseases, such as T2D or coronary heart disease, a large fraction of variance of genetic risk is already known for T1D: indeed, over 50% of the genetic susceptibility to T1D pathogenesis can be explained by risk alleles in the major histocompatibility complex (MHC) region alone, while the remaining genetic contribution has been attributed to variants conferring more moderate risks [27]. The availability of multiple GWAS datasets for T1D, including samples from different geographical sites, genotyped at different locations and on different genotyping platforms (Affymetrix Mapping 500K and Illumina HumanHap550 SNP arrays), allows the unbiased and systematic comparative evaluation of different methods.

## Results

### Overview of the risk assessment algorithms

We tested a machine-learning approach called Support Vector Machine (SVM, see Methods), as well as logistic regression (LR, see Methods) in order to assess individual disease risk for type 1 diabetes (T1D) using three GWAS datasets (Table 1). SVM is one of the most popular classifiers in the field of machine learning and achieves state-of-the-art accuracy in many computational biology applications [28]. In essence, SVM is a supervised machine-learning algorithm that produces a linear boundary to achieve maximum separation between two classes of subjects (cases versus controls), by mathematical transformation (kernel function) of the input features (SNP genotypes) for each subject. Unlike most regression-based methods, SVM allows more input features (such as SNPs or genes) than samples, so it is particularly useful in classifying high-dimensional data, such as microarray gene expression data [29]. We also applied LR as a control algorithm, since it is widely used in genetic studies to model the joint effects of multiple variants. Unlike previous disease assessment studies that typically use genotype data from a handful of validated susceptibility loci, we examined a large ensemble of SNP markers with suggestive evidence for association with T1D, using a few *P*-value cutoff thresholds ranging from 1×10^−3^ to 1×10^−8^, as well as highly stringent quality control measures (see Methods). When more relaxed *P*-value criteria are being used, the contributing SNPs scatter across the genome; when more stringent criteria are used (P<1×10^−8^), only a few independent loci contribute (assuming that all MHC markers represent a single locus). Furthermore, we included the 45 known T1D susceptibility markers [4] into the prediction models to ensure that their predictive values were accounted for. Although these SNP lists may contain some false positive loci that are not genuinely associated with T1D, recent advancements in machine-learning, such as regularization, have made classifiers more tolerant to irrelevant input features [30]. Since we cannot completely eliminate falsely associated loci from the list of predictors, our goal is to include them in the prediction models (using various thresholds) and then assess their influence on performance.

**Table 1:** Description of the three T1D datasets used in the study.

GWAS dataset	Num of Cases	Num of controls	Array platform	Purpose
WTCCC-T1D	1,963	1,480	Affymetrix Mapping 500K	Prediction model training and parameter selection; evaluation of predictive models trained on CHOP/Montreal-T1D
CHOP/Montreal-T1D	1,008	1,000	Illumina HumanHap550	Evaluation of predictive models trained on WTCCC-T1D, using whole-genome imputed genotype data
GoKinD-T1D	1,529	1,458	Affymetrix Mapping 500K	Evaluation of predictive models trained on WTCCC-T1D or CHOP/Montreal-T1D

### Evaluation of risk assessment models by within-study cross-validation

To evaluate the sensitivity of various risk assessment models on the number of predictor variables and the parameters of the models, we performed five-fold cross-validation experiments on the WTCCC-T1D dataset. During each cross-validation, 80% of the samples (both cases and controls) were used to select a subset of SNPs as predictors (see Methods), train a prediction model, and then test on the remaining 20% of the samples. We stress here that the results from a within-study cross-validation do not reflect the true performance of risk assessment (see discussion below), but can help select relevant parameters or thresholds to use. The AUC (area under ROC curve) score was used to evaluate the performance of risk assessment: the value ranges from 0.5 to 1, with a higher number indicating better discriminative power between cases and controls. We found that under various thresholds for SNP selection, the SVM algorithm consistently and slightly out-performed LR, achieving the highest AUC score of ∼0.9 (Table 2). The best performance seems to be achieved when a *P*-value cutoff of 1×10^−5^ is used for selecting SNPs for SVM model training, corresponding to 399–443 SNPs in five cross-validation experiments.

**Table 2:** Evaluation of risk assessment models on the WTCCC-T1D dataset by five-fold cross-validation.

SNP selection	SVM (support vector machine)			LR (logistic regression)			Min #SNP	Max #SNP
	AUC^1^ (SD^2^)	Sensitivity^3^ (SD^2^)	Specificity^3^ (SD^2^)	AUC^1^ (SD^2^)	Sensitivity^3^ (SD^2^)	Specificity^3^ (SD^2^)		
P<1×10^−8^	0.89 (0.017)	0.87 (0.018)	0.75 (0.041)	0.89 (0.016)	0.86 (0.026)	0.75 (0.035)	240	280
P<1×10^−7^	0.89 (0.018)	0.87 (0.024)	0.75 (0.036)	0.88 (0.018)	0.86 (0.034)	0.76 (0.031)	286	328
P<1×10^−6^	0.89 (0.018)	0.88 (0.019)	0.74 (0.041)	0.89 (0.022)	0.86 (0.033)	0.76 (0.044)	328	372
P<1×10^−5^	0.89 (0.013)	0.88 (0.013)	0.73 (0.041)	0.88 (0.014)	0.85 (0.028)	0.75 (0.037)	399	433
P<1×10^−4^	0.88 (0.012)	0.87 (0.021)	0.73 (0.026)	0.87 (0.011)	0.84 (0.016)	0.75 (0.030)	519	558
P<1×10^−3^	0.86 (0.010)	0.85 (0.020)	0.69 (0.015)	0.80 (0.009)	0.77 (0.040)	0.69 (0.025)	1007	1085

1 area under receiver operating characteristic curve.

2 standard deviation.

3 sensitivity and specificity were calculated with default cutoff of zero point.

### Evaluation of risk assessment models on independent datasets

To assess the prediction model in an unbiased way, it is important that independent datasets from different sources be evaluated. This is a practical concern for all GWAS, since the SNPs detected from the training dataset may be spuriously associated with the disease, when cases and controls undergo different DNA preparation protocols [31], when cases and controls are genotyped in different batches, when population stratification is present [32] or when cases share other traits that are unrelated to the disease of interest (for example, cases often have a higher average body mass index than controls when studying T2D [33]). A within-study cross-validation design is not able to adjust for these potential biases which are present in both the training and testing data; therefore, we sought to test the risk assessment models parameterized from the WTCCC-T1D dataset on additional GWAS datasets (Table 1).

Since the CHOP/Montreal-T1D dataset was genotyped using the Illumina platform, we generated whole-genome imputed genotypes using MACH and then utilized shared markers present on the Affymetrix array for the risk assessment. Additionally, we examined a third GWAS dataset from the Genetics of Kidneys in Diabetes consortium (GoKinD), which was genotyped on the same platform as the WTCCC-T1D data, and we used shared markers for the risk assessment. Since this dataset does not contain control subjects, we supplemented this dataset with control subjects from the UK Blood Service (UKBS) collection (a subset of samples from WTCCC not used in the training phase). Similar to the analysis presented above, we varied the *P*-value cutoff thresholds and summarized the results for each threshold.

**Figure 1:**
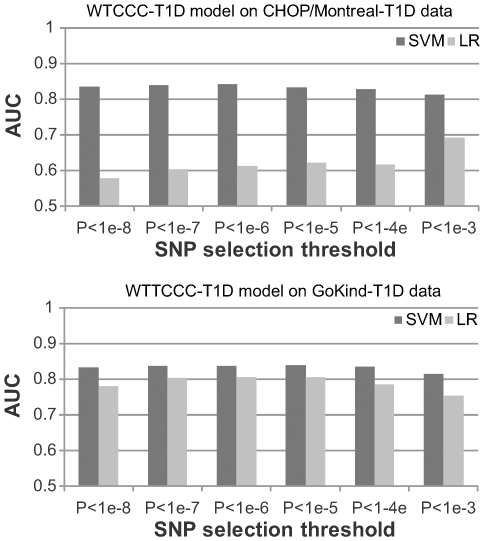
Performance of risk assessment models trained on the WTCCC-T1D dataset. For both the CHOP/Montreal-T1D and the GoKind-T1D datasets, the SVM (support vector machine) algorithm consistently outperforms LR (logistic regression), and the best performance is achieved when SNPs were selected using P-value cutoff of 1×10^−6^ or 1×10^−5^.

We found that the AUC score is 0.83 and 0.84 for the CHOP/Montreal-T1D and GoKinD-T1D datasets, respectively, when SNPs with *P*-value cutoff of 1×10^−5^ were used in the SVM model (Figure 1, Table S1 and Table S2). These values are notably lower than those obtained in cross-validation experiments, suggesting that data differential biases might lead to the inflated performance measures for both SVM and LR seen in Table 2. Nevertheless, SVM consistently achieves higher accuracy than LR, and the AUC scores in both datasets still indicate reasonably good performance. Unlike the previous cross-validation results in Table 2, we found that SVM demonstrate more clear advantage over LR when evaluated by between-study validation. This suggests that SVM may be less susceptible to differential biases than LR through improved utilization of a subset of SNPs, so the differences in performance is less when comparing results generated on independent datasets versus those generated by cross-validation. We also note that the performance advantage of SVM over LR is less obvious, when models were tested on the GoKind-T1D dataset. This could be due to several reasons: First, the control group for the GoKind-T1D dataset was generated at the same site as the WTCCC-T1D dataset, which may introduce differential biases that are shared between the two datasets, with LR being more susceptible to biases than SVM. Second, the CHOP/Montreal-T1D dataset was imputed for proper genotype matching, which may lead to systematic differences from the WTCCC-T1D data from some less well imputed markers due to platform differences. Third, the GoKind-T1D dataset contains markers passing QC in both the WTCCC study and the GoKind study, so they represent a subset of higher-quality markers, making experiments on GoKind-T1D less susceptible to biases. To further investigate this, we re-performed the experiments of training models on WTCCC-T1D and testing on CHOP/Montreal-T1D, using makers that passed QC in GoKind-T1D data (*P*<1×10^−5^ threshold, 409 markers, as opposed to 478 markers): the AUC score for LR increased to 0.82 but remained at 0.84 for SVM, suggesting that inclusion of lower-quality markers led to degraded performance for LR while the impact was less for SVM.

**Figure 2:**
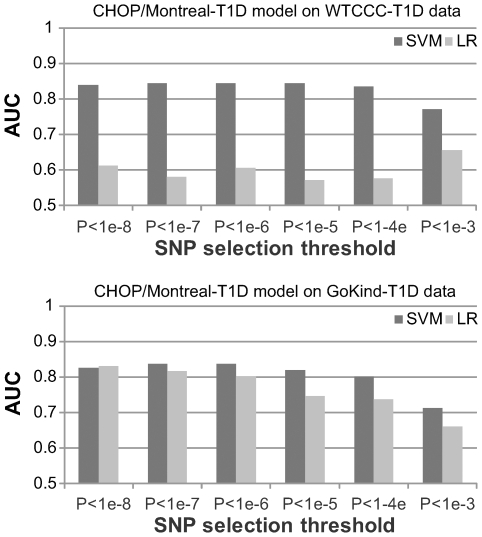
Performance of risk assessment models trained on the CHOP/Montreal-T1D dataset. For both the WTCCC-T1D and the GoKind-T1D datasets, the SVM (support vector machine) algorithm consistently outperforms LR (logistic regression), and the best performance is achieved when SNPs were selected using P-value cutoff of 1×10^−6^ or 1×10^−5^.

Furthermore, we investigated how the algorithms performed when the models were trained on an independent dataset with different size and ascertainment schema. As such, we built prediction models from the imputed CHOP/Montreal-T1D dataset using markers that are present on the Affymetrix arrays, and then evaluated the model performance on the WTCCC-T1D and the GoKind-T1D data (Figure 2, Table S3 and Table S4). Despite the use of a different training dataset, SVM still demonstrated an advantage over LR, with an AUC score of 0.85 on WTCCC-T1D and 0.84 on GoKind-T1D datasets, respectively, when a *P*<1×10^−6^ threshold is used for SNP selection. Altogether, these results suggest that an SVM-based risk assessment algorithm can accommodate differences in training data and can generate consistent, robust results across different datasets.

### Predictive models have high specificity for T1D

Our analyses of three GWAS datasets demonstrate that the SVM-based prediction model is highly reliable in separating T1D cases from control subjects, but a remaining concern is whether the model is specific to T1D, that is, does it tend to predict patients with other diseases as potential T1D cases? To address this concern, we applied the same risk assessment model trained on WTCCC-T1D dataset on six other disease cohorts from WTCCC, including bipolar disorder (BD), coronary heart disease (CAD), Crohn's disease (CD), hypertension (HT), rheumatoid arthritis (RA) and type 2 diabetes (T2D). This analysis is especially interesting, since these diseases include a different subtype of diabetes and two autoimmune diseases (CD and RA), which may share some susceptibility loci with T1D.

**Figure 3:**
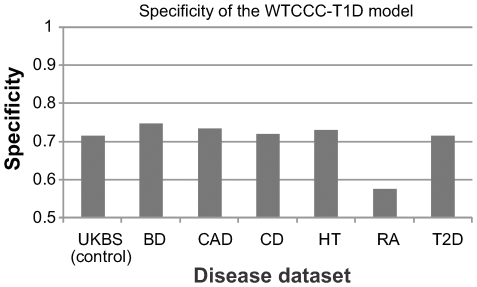
Specificity of the SVM-based risk assessment models. The risk assessment models were parameterized on the WTCCC-T1D dataset and evaluated on other disease cohorts from WTCCC, including bipolar disorder (BD), coronary heart disease (CAD), Crohn's disease (CD), hypertension (HT), rheumatoid arthritis (RA), and type 2 diabetes (T2D). The specificity measure was calculated with default cutoff of zero point. Except for RA, the specificity measures of the prediction model are comparable for other diseases as that for the control subjects.

By testing the SVM-based prediction model for T1D on other six disease cohorts, we found that with the exception of RA, the specificity values are indeed encouraging, ranging from 71.6% for T2D to 74.8% for BD (Figure 3). The specificity for RA, an autoimmune disease with a large genetic susceptibility component from the MHC region, is 57.6%, confirming that T1D and RA do share some genetic risk factors and susceptibility pathways. Besides MHC, the *PTPN22* locus on 1p13 is well known to contribute to both T1D and RA [34],[35], and the WTCCC study reported three additional shared susceptibility loci (*IL2RA* on 10p15, *PTPN2* on 18p11 and chromosome 12q14 region) [36]. For other diseases, these specificity values are at similar range or slightly higher than that for the UK Blood Service (UKBS) control cohort, so a patient affected by diseases unrelated to T1D is not more likely to be predicted as a T1D patient, compared to a control subject. In conclusion, our analyses suggest that the risk assessment model built for T1D is specific to that disease.

### Understanding the behavior of the risk assessment models

To investigate in depth why the SVM algorithm works in the setting of T1D risk assessment, we next evaluated several different forms of the risk assessment models by modifying the predictors or the model parameters. The following six different types of analyses helped us better understand the source of the improved performance of the SVM algorithm.

1. We found that elimination of SNPs in the MHC region severely deteriorate the performance of disease risk assessment. Since a large fraction of the genetic contribution to T1D can be explained by risk alleles in the major histocompatibility complex (MHC) region (∼50%) [37], we tested whether using SNPs outside of the MHC region had any predictive value. This analysis helped identify the relative contribution of MHC-linked SNPs with major effects and other SNPs with moderate effects on disease risks. For this analysis, we removed all the SNPs located from 25 Mb to 34 Mb on chromosome 6: this genomic span is larger than the actual MHC region in order to ensure that SNPs outside MHC but in linkage disequilibrium (LD) with MHC markers are not used in the prediction model. With the *P*<1×10^−5^ threshold, a large proportion of the SNPs (∼83%) were removed from the prediction models. We then tested the performance of the SVM and LR algorithms using the same approach described above. As expected, using SNPs outside of the MHC region do not confer satisfactory performance in disease risk assessment; for example, for the GoKind-T1D data, using SNPs with *P*<1×10^−5^, the AUC score for SVM dropped from 0.84 to 0.64, whereas the AUC score for LR dropped from 0.81 to 0.65. Similar results were obtained for the CHOP/Montreal-T1D dataset. Therefore, elimination of SNPs with major effects severely attenuates the performance of disease risk assessment; on the other hand, our analyses also confirmed that SNPs outside the MHC region do explain a portion of the genetic susceptibility to T1D and can provide complementary information for risk assessment. These results also suggest that whole-genome genotypes provide more information than the costly HLA-typing techniques used in the clinical settings, even for the purpose of risk assessment of MHC-linked diseases such as T1D.
2. We found that pruned sets of independent markers lead to worse performance. The risk assessment model used sets of markers reaching pre-defined thresholds, which may include correlated markers. The SVM algorithm is inherently capable of handling the inter-marker correlation structure, whereas we used regularization techniques [38] in the LR model for addressing this problem. (We did not use stepwise regression model because it is highly unstable when the number of predictor variables is large.) Since many markers are in high LD with each other, we can prune this list to generate a smaller set of markers that have pairwise *r^2^* less than a certain threshold. Intuitively, using fewer markers should lead to information loss and therefore lower predictive power, but we were interested in specifically quantifying this magnitude of loss. We trained a SVM-based prediction models on the WTCCC-T1D dataset using SNPs with *P*<1×10^−5^ that were pruned by various thresholds: when *r^2^* threshold of 0.1, 0.2, 0.5 and 0.8 were used, the AUC scores in the testing dataset were 0.65 (63 SNPs), 0.76 (75 SNPs), 0.79 (153 SNPs) and 0.83 (268 SNPs), respectively. We next used the *P*<1×10^−8^ criteria to select SNP markers, and then performed the same set of computational experiment again: when *r^2^* threshold of 0.1, 0.2, 0.5 and 0.8 were used, the AUC scores in the testing dataset were 0.67 (55 SNPs), 0.76 (60 SNPs), 0.79 (113 SNPs) and 0.82 (184 SNPs), respectively. Altogether, our analyses suggest that the use of independent markers does indeed lose information which is important for risk assessment. Therefore, many previous studies that use only the single most significant SNP per associated loci did not capitalize on all the available genotype information in an optimal manner.
3. We found that radial kernel performs better than linear kernel in SVM. The SVM algorithm that we have used adopted a default radial kernel to transform genotype scores (see Methods), as it is a widely used kernel in most SVM applications. To determine if the data transformation leads to better performance, we also evaluated the SVM algorithm without any transformation, that is, with a linear kernel. Similar to previous experiments, we trained a SVM-based assessment models on the WTCCC-T1D dataset using SNPs with *P*<1×10^−5^. We found that the AUC scores of SVM using linear model are less than those with radial kernel for the GoKind-T1D dataset (0.77 vs 0.84), suggesting that linear combination of predictors (SNPs) is less optimal than higher-order transformation of predictors when separating cases versus controls using SNP genotypes. Similar results were obtained for the CHOP/Montreal-T1D dataset. These observations are consistent with recent findings on the genetic interactions between MHC loci and non-MHC loci for conferring T1D risk [4]. However, unlike LR, the SVM model suffers from poor interpretability, that is, one cannot identify specific pair of SNPs that interact with each other from the model parameters. Additionally, we note that two types of interactions may be important contributors to the risk assessment: statistical interactions between unlinked SNPs (that contribute to liability scale in a non-additive fashion), as well as haplotype interactions (correlated SNPs that are on the same haplotype and are not captured by a model with additive predictors).
4. We investigated the relative effect of modeling correlated SNPs in MHC and non-MHC regions. Our results so far demonstrate that handling of interactions between SNPs, as well as utilizing SNPs with major effects in the MHC region, are important for risk assessment, but their relative contribution is unknown. To test the effect of incorporating non-MHC loci and modeling correlated SNPs for the performance of LR and SVM, respectively, we next performed several variations of the pruned analysis, with the P<1×10^−5^ threshold for selecting SNPs and with pairwise *r^2^*<0.2 threshold for pruning independent sets of SNPs in GoKind-T1D dataset (Table 3). First, we used pruned list of MHC SNPs only, so only independent markers contribute to risk assessment: the AUC for LR and SVM is 0.70 and 0.74, respectively. The decreased performance could be due to the inability to model interaction effects between correlated SNPs, but it also could be due to the (unknown) causal variants being tagged less well in the pruned set. Second, we used pruned list of MHC SNPs plus all non-MHC SNPs: the AUC for LR and SVM is 0.74 and 0.75, respectively, suggesting that additional non-MHC loci contribute to improved performance but the effects are more obvious for LR. Third, we used MHC SNPs only but without pruning: the AUC for LR and SVM is 0.78 and 0.81, respectively, suggesting that both LR and SVM benefit from incorporating correlated SNPs within MHC, which play a more prominent role in the risk modeling than non-MHC markers. Altogether, these analyses suggest that a key contributor to the performance of the SVM algorithm is the better modeling of LD structure among MHC SNPs.
5. We found that an alternative allele coding scheme without assuming genetic model has similar results. In the previous analysis, for each SNP, we coded the three different genotypes (homozygous major allele, heterozygotes, homozygous minor allele) as 0, 1 and 2, respectively. To investigate the sensitivity of prediction models on allele coding, we next explored an alternative coding scheme, by generating two dummy variables (0 or 1) for each SNP, indicating the presence or absence of an allele. This coding scheme effectively doubles the number of predictor variables, but without assuming an additive risk model for each SNP. We tested the new coding scheme on the GoKind-T1D dataset, and found that the AUC score remained the same as 0.84. For the CHOP/Montreal-T1D dataset, the AUC Score slightly decreased from 0.83 to 0.82. Therefore, relaxing genetic model assumptions do not appear to have a major impact on the performance of risk models.
6. We found that the collection of known T1D susceptibility loci has poor performance. Recent progress with GWAS has enabled the identification of dozens of confirmed and replicated T1D susceptibility loci [3],[4]. As a negative control experiment, we tested the performance of risk assessment using only established susceptibility loci. This analysis is similar to the several previously published T2D risk assessment studies, in that the prediction model only considers known information, while ignoring other potentially associated loci. We built risk assessment models around the WTCCC-T1D dataset, using 45 known T1D susceptibility SNPs compiled from a recent meta-analysis [4], after excluding one locus on chromosome X (Table S5). Note that only one representative SNP from the MHC region is used in the assessment models. For the SVM algorithm, the AUC scores are 0.66 for the GoKind-T1D dataset and 0.65 for the CHOP/Montreal-T1D dataset, indicating a limited value of risk assessment using a reduced number of validated SNPs. For the LR algorithm, the AUC scores are 0.68 for both the GoKind-T1D and the CHOP/Montreal-T1D datasets, which are slightly higher than those obtained using the SVM algorithm. Nevertheless, the relatively modest performance is not unexpected, and echoes what has already been observed in T2D disease assessment studies. Collectively, this analysis confirms that one of the keys to success is the use of a large ensemble of loci associated to the disease of interest, at the cost of including potential false positive loci.

**Table 3:** Comparative analysis of prediction models by including different sets of markers.

Marker selection (P<1×10^−5^)	# markers	AUC^1^ (LR)	AUC^1^ (SVM)
All (MHC and non-MHC) SNPs	409	0.81	0.84
MHC SNPs	338	0.78	0.81
Non-MHC SNPs	71	0.65	0.64
Pruned MHC and non-MHC SNPs^2^	82	0.74	0.76
Pruned MHC SNPs^2^	27	0.70	0.74
Pruned MHC SNPs^2^ and not pruned non-MHC SNPs	98	0.74	0.75

1 area under receiver operating characteristic curve.

2 SNPs are pruned using pairwise *r^2^* threshold of 0.2.

## Discussion

In this study, we tested the plausibility of building a classifier and using a large number of SNPs for disease risk assessment on three large T1D datasets. In general, the SVM algorithm achieved satisfactory performance when hundreds of SNPs were included in prediction models, with AUC scores of ∼0.84 for predicting disease risk for T1D in several GWAS datasets. In contrast, the SVM or the LR algorithm achieved only an AUC score of 0.66–0.68 when 45 known T1D susceptibility loci were used. This difference clearly indicates that the predictive value lies in utilizing a large number of SNPs in a sophisticated machine-learning algorithm. We note that another recent study also reported that using thousands of SNPs improve the performance of disease risk assessment compared to using fewer SNPs for diseases studied by WTCCC [39], although the study used a cross-validation design. On the other hand, we observed a decrease in the predictive accuracy when too many SNPs were used, suggesting an upper bound of the number of SNPs for T1D risk assessment before noises from falsely associated markers lead to degraded performance. However, we caution that this upper bound depends on the sample size and the power of the study to rank truly associated SNPs higher than background noises.

One of the major differences between the two classifiers used in the study is in their capability of handling main and interaction effects. SVM takes into account both effects, while LR aims to model linear main effects but ignores interaction. As an example, for a simple interaction model with two risk loci A and B, the disease risk will increase significantly only if A = 0 and B = 1 or A = 1 and B = 0. Such interaction can be captured by SVM using various kernels, but not by the simple LR model. In our study, we observed that SVM outperformed LR by taking into account both main and interaction effects, implying that both genes and their interactions contribute to T1D. This is particularly important for T1D, as many previous studies have already shown that risk from MHC and non-MHC regions accumulates at a rate less than expected from the model of multiplicative effects, and that the relative risks for non-MHC loci are reduced when MHC-related risk is high [4]. Additionally, we also found that better modeling of LD structure within MHC (haplotype interactions) play a major role in SVM-based risk modeling. However, these issues have been largely ignored in previous simulation studies on disease risk assessment [13],[15],[40], probably because the appropriate modeling of interaction effect is by itself not well understood. Nevertheless, some previous real-data studies already documented the importance of interactions effects in prediction model for quantitative or qualitative phenotypes: for example, Lee *et al* have applied a MCMC approach that takes into account of within and between loci interactions, for phenotype prediction in heterogeneous stock mouse population in a cross-validation design [41]. Therefore, while simulation studies are useful in drawing general qualitative conclusions such as that predictive accuracy increases with heritability, their quantitative findings may not be accurate because of the “non-interaction” assumptions they have to make. Although we have limited understanding of the interaction patterns of variants underlying common and complex diseases, we argue that the previous simulation results may not necessarily reflect real-world scenario.

Although the implementation of individual risk assessment in clinical settings could have major economic benefit to the public health at the population level [42], the clinical utility of individual risk assessment depends on a few factors that must be taken into account. First, the appropriateness of individual risk assessment is dependent on the genetic etiology of the disease [16]. Given that the vast majority of the phenotypic variation in T1D can be explained by genetic factors, T1D assessment would have a strong basis for being applied in clinical settings: in fact, HLA-typing, albeit being both costly and imperfect, is now being used in clinical settings for assessing T1D risk for siblings of affected patients. Unlike T1D, where genetic factors are estimated to explain ∼90% of the phenotypic variance, the heritability estimates for T2D are less than 50% [24]. Therefore, even if all genetic risk factors are identified ultimately for T2D and a perfect SNP-based prediction model is available for the disease, they would have less impact in a clinical setting than T1D prediction models.

Second, the clinical utility of a risk assessment model depends on the disease prevalence at the particular clinical setting. Using the sensitivity and specificity measures for the WTCCC-T1D model on CHOP/Montreal-T1D datasets (Figure S1), we evaluated three scenarios of diagnostic testing using SNP arrays: (1) general population (disease prevalence = 0.4%), (2) siblings of affected patients (disease prevalence = 6%), (3) siblings of early-onset patients who developed diabetes before 5 yrs of age (disease prevalence = 13%). When a general population is screened by the prediction model, the positive predictive values are relatively modest, indicating that the risk assessment model is not of much utility for population-level screening. However, in a realistic clinical setting, where siblings of affected patients are evaluated, the WTCCC-T1D prediction model achieves a positive predictive value of 16% and a negative predictive value of almost 100%; that is, ∼16% of predicted positive patients will eventually develop the disease, while very few predicted negative patients will develop the disease, with overall accuracy of 93%. Finally, for siblings of early-onset patients, the positive predictive value reaches 31%, while a strong negative predictive value of 96% can still be retained with an overall prediction accuracy of 87%. Although T1D has a large genetic contribution from risk alleles in the MHC region, it is well known that costly HLA-typing *per se* is not sufficient for T1D risk assessment with high accuracy. Based on our results, we envision that low-cost SNP genotyping platforms may have the potential to replace HLA-typing in assessing T1D risk in clinically relevant settings.

Third, for a given disease, the best assessment model and the most optimal number of predictors (SNPs) may depend on the distribution of effect sizes. Some autoimmune diseases, such as T1D, have major-effect loci (MHC) that explain a large proportion of the genetic risk (∼50% for T1D), with additional moderate-effect loci that explain the remaining proportion. Therefore, for these diseases, the collection of SNPs with *P*<1×10^−5^ in a given GWAS probably already captured the vast majority of the variance for genetic susceptibility, and can lead to good prediction performance. Therefore, diseases such as T1D might represent an extreme example where genotype-based risk assessment is clinically feasible. In contrast, MHC loci play a much less important role or no role in CD or T2D susceptibility, so a much more liberal *P*-value threshold may be required for SNP selection, to ensure the capture of a large fraction of the genetic risk in prediction models. This step will likely include more markers that are falsely associated with the disease in prediction models, and may dilute the contribution from genuinely associated loci. Taking interception from independent datasets (for example, SNPs with *P*<0.05 in two GWAS) may be explored for risk assessment on these diseases. Furthermore, diseases such as psychiatric disorders do not appear to even have any major-effect loci that are common, so accurate assessment of disease risk may require even more markers or whole-genome markers.

Finally, besides genetic factors, other risk factors that are specific for each disease need to be accounted for in order to make an accurate risk assessment. Early onset diseases such as T1D may be less dependent on non-genetic factors. In contrast, T2D is a late-onset disease with a range of known environmental risk factors contributing to its pathogenesis, and may be predicted more accurately if such factors are also used. Therefore, a comprehensive disease risk assessment model should try to take into account environmental risk factors, such as diet and smoking habits, as well as other predictor variables such as gender and BMI in order to improve performance. These factors are most likely disease-specific and can be identified from cumulative epidemiological studies on each disease. We note that the SVM model used in our study can readily take into account additional predictor variables.

In conclusion, the results from recent GWAS have yielded enormous amounts of data that can be mined and utilized for better understanding of human disease, including disease risk assessment using genetic profiles. Although only a small fraction of risk factors for complex diseases have been identified to date, other variants with moderate effects are present in the GWAS data, and a risk assessment algorithm should be able to take advantage of these variants for improved performance. We expect that methods that utilize whole-genome data, rather than a few “validated” susceptibility loci, could improve predictive accuracy and have greater impact on health care in the future; for example, by applying personalized intervention strategies on newborns who are at risk of developing T1D, we may reduce their risk of developing the disease or be better prepared to treat the disease. This would be feasible if these individuals can be identified from genetic risk profiles, using algorithms (such as the SVM algorithm proposed in this study) with high positive predictive values.

## Methods

### Description of study subjects

Type 1 Diabetes (T1D) GWAS dataset from WTCCC: We accessed the 500K Affymetrix chip genotype data from WTCCC on ∼1,500 samples from the 1958 British Birth Cohort, ∼1,500 samples from the UK Blood Service Control Group, as well as ∼2,000 samples each from the following disease collections: type 1 diabetes (T1D), type 2 diabetes (T2D), rheumatoid arthritis (RA), inflammatory bowel disease (IBD), bipolar disorder (BD), hypertension (HT), coronary artery disease (CAD), as previously described [36]. For each dataset, we have downloaded the genotype calls generated by the Chiamo algorithm, and we have applied the default confidence score of 0.9 to keep the high-quality genotype calls. In addition, we removed the 30,956 SNP markers failing QC threshold (due to one of three criteria), as specified in the website. For each dataset, we observed the same recommended sample exclusion criteria, as specified in the various “exclusion-list” files in the data repository.

T1D GWAS dataset from GoKinD: The Genetics of Kidneys in Diabetes (GoKinD) study [43],[44] T1D case data were downloaded from dbGaP [45]. This dataset consists of T1D cases only (about half have diabetic nephropathy but half without nephropathy). Therefore, we subsequently used the UK Blood Service dataset from WTCCC as control subjects for the risk assessment sensitivity/specificity analysis. Both the case and control genotypes in this dataset were independent and not used in the prediction model building.

T1D GWAS datasets from CHOP and Montreal: The third T1D case series used in our study was genotyped at the Children's Hospital of Philadelphia (CHOP), and a subset of this cohort has been previously described [46]. The dataset contains 1,008 T1D subjects and 1,000 control subjects. The T1D families and cases were identified through pediatric diabetes clinics at the Children's Hospital of Montreal and at CHOP. All control subjects were recruited through the Health Care Network at CHOP. The multi-dimensional scaling analysis on genotype data was used to identify subjects of genetically inferred European ancestry. All subjects were genotyped at ∼550,000 SNPs by the Illumina HumanHap550 Genotyping BeadChip; to apply the prediction model on these subjects, we subsequently used genotype imputation (see below) to generate imputed genotypes on these subjects.

### Genotype imputation on Illumina arrays

We used the Markov Chain Haplotyping (MACH) software (http://www.sph.umich.edu/csg/abecasis/MaCH/index.html) for genotype imputation on markers that are present in the Affymetrix array from WTCCC, but not present in the Illumina HumanHap550K arrays used by us. The default two-step imputation procedure is adopted for imputation: (1) In the first step, 500 randomly selected subjects of European ancestry are used to estimate the best model parameters. This model includes both an estimate of the “error” rate for each marker (an omnibus parameter which captures both genotyping error, discrepancies between the imputed platform and the reference panel, and recurrent mutation) and of “crossover” rates for each interval (a parameter that describes breakpoints in haplotype stretches shared between the imputed and the reference panel). The software requires several input files for SNPs and phased haplotypes; we used the HapMap phased haplotypes (release 22) on CEU subjects, as downloaded from the HapMap database (http://www.hapmap.org). (2) In the second step, we used the optimized model parameters to impute the genotypes on >2 million SNP markers in HapMap data. The default Rsq threshold of 0.3 in the mlinfo file was used to flag unreliable markers used in the imputation analysis, and the posterior probability threshold of 0.9 was used to flag unreliable genotype calls. The imputed genotype data were then checked for strand orientation (since the Affymetrix genotype data from WTCCC may not align correctly with the HapMap phased genotype data) and inconsistencies were resolved using the flip function in the PLINK software [47].

### Disease risk assessment model building

For our purposes, genetic profiles on *p* SNPs for *n* individuals may be summarized by an n*p matrix **G** = (*g_ij_*), where *g_ij_* denotes the genotypic value of SNP *i* in individual *j*. The genotype data are encoded by 0, 1 and 2. In genome wide association studies, data for each individual consist of a genetic profile **G** ***_i_*** = (*g_i1_*,…, *g_ip_*) and a disease indicator *y_i_*∈{0,1}, namely, we have predictor variables **G** ***_i_*** and response variable *y_i_*. Based on current genotyping technologies, the number of SNPs *p* typically can be as large as several hundred thousands, whereas the number of individuals *n* is several thousands in typical genetic studies. Therefore, in our comparison of prediction methods, we use only the list of markers reaching a pre-defined statistical threshold of association with disease. As a result, the number of SNPs used for disease prediction is substantially reduced to at most one or two thousands in our studies.

In machine learning, a predictor or classifier is built from past experience and is used to make predictions of unknown future. In our case, a trained classifier partitions the space of genetic profiles into two disjoint and exhaustive subsets, cases and controls, such that for a DNA sample with genetic profile **g** = (*g_1_*,…, *g_p_*), the classifier can accurately predict if it is a case sample or a control sample. With the large amount of WTCCC case-control data available for training, we were able to build a predictor with good accuracy that we subsequently applied on two independent test cohorts. While the challenges ahead in translating the emerging genomic knowledge into clinical practice, we envision the approach we have taken to be an important step towards these goals.

Many classification methods have been developed and applied to various domains. No classifier could show dominant performance consistently in all applications. In our comparison study, we compared the efficacy of two representative ones, logistic regression (LR) and support vector machine (SVM). A logistic regression model and its variants are one of the most widely used approaches in genetic data analysis. They are simple but often provide an adequate and interpretable description of how the inputs affect the output. In addition, simpler linear methods work as well or better for microarray classification problems where the number of candidate predictors exceeds the number of samples by orders of magnitude [48]. We consider the logistic regression model, which models the posterior probabilities of being a case or a control via a linear combination of *g_i1_*,…, *g_ip_*. Formally,
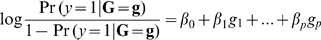
Under the LR model (*β_0_*,*β_1_*,…,*β_p_*), the probability of being cases (*y* = 1) for a genetic profile is exp(**β** ^T^ **g**)/(1+exp(**β** ^T^ **g**)), where **β** = (*β_0_*, *β_1_*,…, *β_p_*) and **g** = (*1*, *g_1_*,…, *g_p_*)^T^. Given the training data, the LR model is fit to get a maximum likelihood estimate (MLE) of **β**, and this estimate can be used for future prediction. It is noted that *g_i_*∈{0,1,2} is treated as a numeric value.

The LR model has the advantage that the main effect of each SNP to the phenotypes has a linear and interpretable description. The effect of each SNP can be naturally interpreted as the increase of the log odds ratio in favor of being a case when the count of risk allele changed by 1. One caveat of using LR model in GWAS is that linkage disequilibrium dependency of input markers may make the parameter estimation unstable. To address this issue, we imposed a L∧2 regularization on the LR model building [38]. We implemented our LR model based on the *stepPlr* package in R developed by Park and Hastie [49].

The second classifier that we have applied is the support vector machine (SVM) algorithm [26],[50]. SVM is one of the most popular supervised classifiers in the field of machine learning and has been widely used in many bioinformatics applications. SVM aims to find an optimal separating hyperplane between cases and controls and this is achieved by two key factors: large margin separation and kernel functions. The classification problem is formulated by SVM as the optimization problem
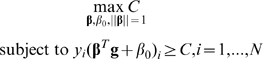
When the samples are linearly separable, the optimal hyperplane is the one that creates the biggest *margin C* between the training points for cases and controls. When the samples in feature space (*g_1_*,…, *g_p_*) are not linearly separable, certain overlap can be allowed by introducing the slack variable 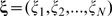 and the constraint is modified as 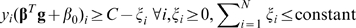. More importantly, linear separability can be obtained in an expanded input feature space h(**g**) by using kernel functions. Specially for SVM, the explicit transformation h(**g**) is not needed and only knowledge of the kernel function is required *K*(**g**, **g**′) = <h(**g**), h(**g′**)>, which computes inner products in the expanded feature space. Two popular kernel functions in the SVM literature are
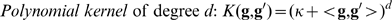


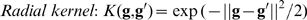
When 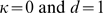, it is a special case of the polynomial kernel called the *linear kernel*, which operates in the original input feature space. Using non-linear kernel functions, SVM can produce nonlinear boundaries to separate two classes of objects by constructing equivalent linear boundaries in an expanded input feature space. Such transformations usually increase accuracy considerably on one hand, but on the other hand the transformations cause poor interpretability, namely, it is not clear how the inputs affect the output even though high accuracy is obtained.

In the case of disease risk assessment, SVM constructs an optimal linear boundary (prediction model) in an expanded input feature space (in our case, transformed genotype calls for a collection of SNPs). New features, or a transformation of input features (SNP genotypes), can be derived by using the kernel function [50], with the goal of making inputs linearly separable. However, no biological interpretation can be attached to each predictor variable (SNP) in the prediction model. We implemented the SVM model using the machine learning package *e1071* in R. It is based on the popular SVM library LIBSVM [51]. For model building, we used all default options including the radial kernel. To assess the effect of data transformation implemented in the radial kernel, we have also explored the use of the linear kernel and compared their predictive performance.

### SNP data processing and coding

For the case-control datasets, to reduce the potential concern on stratification or batch effects, we applied highly stringent quality control measures to select SNPs to use in the prediction models. We applied several quality filters, including call rate >95%, minor allele frequency>5% and Hardy-Weinberg equilibrium P-value >1×10^−3^, for the selection of SNPs. We used the EigenStrat algorithm [52] on genotype data, and selected subsets of SNPs reaching pre-defined P-value thresholds to build prediction models, including P<1×10^−8^, P<1×10^−7^, P<1×10^−6^, P<1×10^−5^, P<1×10^−4^ and P<1×10^−3^. Additionally, only autosomal markers were used in our prediction model so that the model can be applied to both genders. Finally, we removed SNPs from the training data that are not present in the testing data (for example, SNPs not in HapMap or SNPs without known dbSNP identifiers). Genotypes with missing values were imputed by sampling from the allele frequency distribution. We coded homozygous major allele, heterozygotes and homozygous minor allele as 0, 1 and 2, respectively.

### Performance evaluation

The simplest and most widely used method for estimating prediction error may be *K*-fold cross-validation. However, due to the data differential biases discussed in the paper, we caution that cross-validation approach may severely inflate the true predictive value. In *K*-fold cross-validation, the data is split into *K* roughly equal-sized parts; for the *k*th part of the data, the classifier is trained based on the other *K*-1 parts of the data and then used to predict the *k*th part of the data. The process is iterated for *k* = 1, 2, …, *K* and predictions for all data are obtained. The predictions are then used for estimating prediction performance of the classifier. Typical choices of *K* are 5 or 10. We do five-fold cross-validation to compare performance of the two classifiers over the seven case-control disease datasets. Specifically, we measure accuracy, sensitivity and specificity defined as follows,
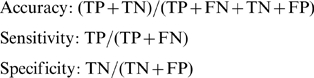
where TP, TN, FP and FN denote the number of true positives, true negatives, false positives and false negatives, respectively. Note that since prediction algorithms typically give quantitative assessment, we used the default cutoff of zero point for the sensitivity and specificity calculation.

In addition, we also evaluated the performance by the area under receiver operator characteristic (ROC) curve scores (AUC scores). ROC is a widely used means to evaluate the discrimination ability of binary classification methods when the test results are continuous measures. ROC curves display the relationship between sensitivity (true positive rate) and 1-specificity (false positive rate) across all possible threshold values that define the positivity of a condition (in our case, whether a subject has T1D diagnosis). The AUC scores may range from 0.5 to 1, with a higher score indicating better discriminatory power.

Furthermore, to measure the performance of a prediction model in clinical settings, we calculated the positive predictive value (PPV) and negative predictive value (NPV), which incorporate the disease prevalence in the testing population. These two values were calculated as:
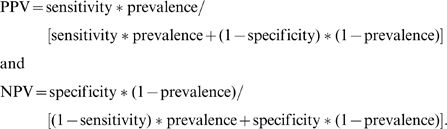



## Supporting Information

Figure S1 Illustration on how positive predictive value (PPV) and negative predictive value (NPV) vary with respect to disease prevalence in a testing population. The figure is based on sensitivity and specificity estimates from WTCCC-T1D data set on CHOP-T1D data when P<1×10^−5^ is used. The three vertical lines represent three different scenarios of clinical testing, with disease prevalence of 0.4%, 6%, and 13%, respectively.
(0.02 MB TIF)

Table S1 Prediction performance of the WTCCC-T1D trained model on the CHOP/Montreal-T1D datasets.
(0.02 MB PDF)

Table S2 Prediction performance of the WTCCC-T1D trained model on the GoKind-T1D datasets.
(0.02 MB PDF)

Table S3 Prediction performance of the CHOP/Montreal-T1D trained model on the WTCCC-T1D datasets.
(0.02 MB PDF)

Table S4 Prediction performance of the CHOP/Montreal-T1D trained model on the GoKind-T1D datasets.
(0.02 MB PDF)

Table S5 A list of 46 previously validated T1D susceptibility loci reported in the meta-analysis by Barrett et al.
(0.12 MB PDF)
