# Supplementary figures and images for: From Disease Association to Risk Assessment: An Optimistic View from Genome-Wide Association Studies on Type 1 Diabetes
Source: PLoS Genet. 2009 Oct 9;5(10):e1000678. doi: 10.1371/journal.pgen.1000678 (PMC2748686; doi:10.1371/journal.pgen.1000678)

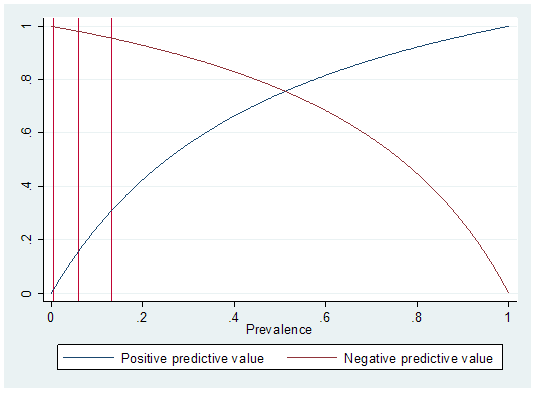

Supplement: Figure S1 — Illustration on how positive predictive value (PPV) and negative predictive value (NPV) vary with respect to disease prevalence in a testing population. The figure is based on sensitivity and specificity estimates from WTCCC-T1D data set on CHOP-T1D data when P<1×10^−5^ is used. The three vertical lines represent three different scenarios of clinical testing, with disease prevalence of 0.4%, 6%, and 13%, respectively. (0.02 MB TIF) [file pgen.1000678.s001.tif]
