# Supplementary material for: From Disease Association to Risk Assessment: An Optimistic View from Genome-Wide Association Studies on Type 1 Diabetes
Source: PLoS Genet. 2009 Oct 9;5(10):e1000678. doi: 10.1371/journal.pgen.1000678 (PMC2748686; doi:10.1371/journal.pgen.1000678)
Supplement: Table S1 — Prediction performance of the WTCCC-T1D trained model on the CHOP/Montreal-T1D datasets. (0.02 MB PDF) [file pgen.1000678.s002.pdf]

| Algorithm | P Cutoff | $1x10^{-8}$ | $1x10^{-7}$ | $1x10^{-6}$ | $1x10^{-5}$ | $1x10^{-4}$ | $1x10^{-3}$ |
|---|---|---|---|---|---|---|---|
| | #SNPs | 312 | 365 | 417 | 478 | 632 | 1145 |
| SVM | AUC | 0.835 | 0.84 | 0.841 | 0.833 | 0.828 | 0.812 |
| | Sensitivity | 0.776 | 0.77 | 0.761 | 0.772 | 0.767 | 0.765 |
| | specificity | 0.758 | 0.762 | 0.758 | 0.74 | 0.75 | 0.702 |
| LR | AUC | 0.58 | 0.605 | 0.613 | 0.622 | 0.617 | 0.693 |
| | Sensitivity | 0.517 | 0.536 | 0.549 | 0.546 | 0.551 | 0.599 |
| | specificity | 0.588 | 0.604 | 0.601 | 0.62 | 0.588 | 0.686 |
