# Supplementary material for: From Disease Association to Risk Assessment: An Optimistic View from Genome-Wide Association Studies on Type 1 Diabetes
Source: PLoS Genet. 2009 Oct 9;5(10):e1000678. doi: 10.1371/journal.pgen.1000678 (PMC2748686; doi:10.1371/journal.pgen.1000678)
Supplement: Table S2 — Prediction performance of the WTCCC-T1D trained model on the GoKind-T1D datasets. (0.02 MB PDF) [file pgen.1000678.s003.pdf]

Algorithm	P Cutoff	$1x10^{-8}$	$1x10^{-7}$	$1x10^{-6}$	$1x10^{-5}$	$1x10^{-4}$	$1x10^{-3}$
	#SNPs	267	313	358	409	539	1004
SVM	AUC	0.832	0.838	0.837	0.839	0.834	0.814
	Sensitivity	0.779	0.792	0.793	0.791	0.788	0.795
	specificity	0.716	0.719	0.716	0.717	0.708	0.669
LR	AUC	0.781	0.803	0.805	0.805	0.786	0.753
	Sensitivity	0.708	0.734	0.732	0.723	0.716	0.707
	specificity	0.679	0.695	0.706	0.709	0.713	0.663
