# Supplementary material for: From Disease Association to Risk Assessment: An Optimistic View from Genome-Wide Association Studies on Type 1 Diabetes
Source: PLoS Genet. 2009 Oct 9;5(10):e1000678. doi: 10.1371/journal.pgen.1000678 (PMC2748686; doi:10.1371/journal.pgen.1000678)
Supplement: Table S3 — Prediction performance of the CHOP/Montreal-T1D trained model on the WTCCC-T1D datasets. (0.02 MB PDF) [file pgen.1000678.s004.pdf]

Algorithm	P Cutoff	$1x10^{-8}$	$1x10^{-7}$	$1x10^{-6}$	$1x10^{-5}$	$1x10^{-4}$	$1x10^{-3}$
	#SNPs	192	235	280	340	445	1098
SVM	AUC	0.839	0.844	0.845	0.843	0.834	0.771
	Sensitivity	0.876	0.876	0.864	0.855	0.833	0.724
	specificity	0.632	0.646	0.653	0.666	0.667	0.676
LR	AUC	0.614	0.582	0.607	0.573	0.578	0.655
	Sensitivity	0.616	0.591	0.611	0.552	0.554	0.581
	specificity	0.535	0.507	0.536	0.532	0.551	0.629
