# Supplementary material for: From Disease Association to Risk Assessment: An Optimistic View from Genome-Wide Association Studies on Type 1 Diabetes
Source: PLoS Genet. 2009 Oct 9;5(10):e1000678. doi: 10.1371/journal.pgen.1000678 (PMC2748686; doi:10.1371/journal.pgen.1000678)
Supplement: Table S4 — Prediction performance of the CHOP/Montreal-T1D trained model on the GoKind-T1D datasets. (0.02 MB PDF) [file pgen.1000678.s005.pdf]

**Algorithm**	**P Cutoff**	**1x10^{-8}**	**1x10^{-7}**	**1x10^{-6}**	**1x10^{-5}**	**1x10^{-4}**	**1x10^{-3}**
	**#SNPs**	166	201	237	295	392	998
**SVM**	**AUC**	0.826	0.837	0.838	0.819	0.801	0.713
	**Sensitivity**	0.83	0.825	0.815	0.78	0.758	0.611
	**specificity**	0.688	0.697	0.709	0.711	0.711	0.687
**LR**	**AUC**	0.83	0.816	0.802	0.747	0.737	0.66
	**Sensitivity**	0.788	0.806	0.754	0.695	0.689	0.532
	**specificity**	0.709	0.682	0.689	0.671	0.66	0.666
