# Supplementary material for: From Disease Association to Risk Assessment: An Optimistic View from Genome-Wide Association Studies on Type 1 Diabetes
Source: PLoS Genet. 2009 Oct 9;5(10):e1000678. doi: 10.1371/journal.pgen.1000678 (PMC2748686; doi:10.1371/journal.pgen.1000678)
Supplement: Table S5 — A list of 46 previously validated T1D susceptibility loci reported in the meta-analysis by Barrett et al. (0.12 MB PDF) [file pgen.1000678.s006.pdf]

Barrett et al SNP	WTCCC SNP	r2	Chr	LD region	Gene or Num of genes
rs2269241	rs2269241	1	1p31.3	63.87–63.94	*PGM1*
rs2476601	rs6679677	1	1p13.2	113.62–114.46	*PTPN22*
rs2816316	rs1323296	1	1q31.2	190.73–190.82	*RGS1*
rs3024505	rs3024505	1	1q32.1	204.87–205.12	*IL10*
rs1534422	rs10175906	0.72	2p25.1	12.53–12.60	*(0)*
rs917997	rs2041756	1	2q12.1	102.22–102.58	*IL18RAP*
rs1990760	rs7608315	0.59	2q24.2	162.67–163.10	*IFIH1*
rs3087243	rs3087243	1	2q33.2	204.38–204.53	*CTLA4*
rs11711054	rs6441961	1	3p21.31	45.96–46.63	*CCR5*
rs10517086	rs17698094	0.54	4p15.2	25.64–25.75	*(0)*
rs4505848	rs17388568	0.83	4q27	123.13–123.83	*IL2*
rs6897932	rs6897932	1	5p13.2	35.84–36.07	*IL7R*
rs9268645	rs9268645	1	6p21.32	24.70–34.00	*MHC*
rs11755527	rs11755527	1	6q15	90.86–91.10	*BACH2*
rs9388489	rs9388489	1	6q22.32	126.48–127.46	*C6orf173*
rs2327832	rs2327832	1	6q23.3	137.80–138.40	*TNFAIP3*
rs1738074	rs2016588	0.57	6q25.3	159.13–159.62	*TAGAP*
rs7804356	rs10486478	1	7p15.2	26.62–27.17	*(10)*
rs4948088	rs10486748	0.41	7p12.1	50.87–51.64	COBL
rs7020673	rs7020673	1	9p24.2	4.22–4.31	*GLIS3*
rs12251307	rs12251307	1	10p15.1	6.07–6.24	*IL2RA*
rs11258747	rs11258747	1	10p15.1	6.48–6.59	*PRKCQ*
rs10509540	rs11816865	0.66	10q23.31	90.00–90.27	*C10orf59*
rs7111341[1]	rs6578252	0.07	11p15.5	2.02–2.26	*INS*
rs4763879	rs4763879	1	12p13.31	9.51–9.87	*CD69*
rs2292239	rs2292239	1	12q13.2	54.64–55.09	*ERBB3*
rs1678536	rs1678542	0.62	12q13.3	55.27–56.82	*Multiple*
rs3184504	rs1265566	0.25	12q24.12	109.77–111.72	*SH2B3*
rs1465788	rs1465788	1	14q24.1	68.24–68.39	*(2)*
rs4900384	rs4900384	1	14q32.2	97.43–97.60	*(0)*
rs3825932	rs3825932	1	15q25.1	76.77–77.05	*CTSH*
rs12708716	rs12708716	1	16p13.13	10.92–11.56	*CLEC16A*
rs12444268	rs12444268	1	16p12.3	20.17–20.28	*(2)*
rs4788084	rs151181	0.9	16p11.2	28.19–28.94	*IL27*
rs7202877	rs4536500	0.64	16q23.1	73.76–74.09	*(7)*
rs16956936	rs16956936	1	17p13.1	7.56–7.66	*(2)*
rs2290400	rs1008723	1	17q12	34.63–35.51	*ORMDL3*
rs7221109	rs7221109	1	17q21.2	35.95–36.13	*(3)*
rs1893217	rs16939895	0.69	18p11.21	12.73–12.92	*PTPN2*
rs763361	rs1788101	0.18	18q22.2	65.63–65.72	*CD226*

rs425105	rs4804000	0.69	19q13.32	51.84–52.02	*(5)*
rs2281808	rs2281808	1	20p13	1.44–1.71	*(3)*
rs11203203	rs11203203	1	21q22.3	42.68–42.76	*UBASH3A*
rs229541	rs229541	1	22q13.1	35.90–36.00	*C1QTNF6*
rs5753037	rs41176	1	22q12.2	28.14–29.00	*(14)*
rs2664170[2]			Xq28	153.48–154.10	*(16)*

1: The *INS* locus is not well covered by the Affymetrix array.
2: The chromosome X marker is not used in our study.
